# Clinical and Morphological Bone Marrow Characteristics of Pearson Syndrome: About Three Consecutive Cases and Review of the Literature

**DOI:** 10.1155/crpe/3076141

**Published:** 2025-05-18

**Authors:** Gaetan-Nagim Degroot, Aurélie Empain, Laurence Dedeken, Anne Demulder, Laurence Rozen

**Affiliations:** ^1^Laboratory of Hematology, Brussels University Hospital Laboratory (LHUB), Université Libre de Bruxelles (ULB), Brussels, Belgium; ^2^Laboratory of Hematology, CHU Brugmann University Hospital, Université Libre de Bruxelles (ULB), Brussels, Belgium; ^3^Department of Paediatrics, Metabolic and Nutrition Unit, Division of Endocrinology, Diabetes and Metabolism, Queen Fabiola Children's University Hospital (HUDERF)–H.U.B., Metabolic Centre ULB-VUB, Brussels, Belgium; ^4^European Reference Network for Hereditary Metabolic Disorders (MetabERN) Member, Padova, Italy; ^5^Department of Paediatrics, Hematology-Oncology Department, Queen Fabiola Children's University Hospital (HUDERF)–H.U.B., Université Libre de Bruxelles (ULB), Brussels, Belgium

**Keywords:** bone marrow characteristics, clinical features, hyporegenerative anemia, mitochondrial DNA deletion, Pearson's syndrome

## Abstract

Pearson syndrome (PS) is a rare and fatal multisystem disorder caused by a mitochondrial DNA (mtDNA) deletion. Most patients develop refractory anemia in early infancy, rapidly followed by multiple complications such as failure to thrive, muscle hypotonia, pancreatic insufficiency, and renal tubulopathy. Although the definitive diagnosis is established by mtDNA sequencing, bone marrow (BM) cytology is a cornerstone of diagnosis, typically revealing precursor vacuolization and ring sideroblasts. We report here three cases of patients with PS encountered in our institution and summarize the clinical and hematological features of PS through a systematic review of the literature. The first symptoms mostly appear during the first month of life and rarely after 18 months. Hyporegenerative anemia, a hallmark of the disease, is the most common initial symptom, followed at a distance by neutropenia and thrombocytopenia. Gastrointestinal and metabolic symptoms such as failure to thrive and lactic acidosis are the most frequent non-hematological symptoms, even in the rare cases without hyporegenerative anemia. Vacuolization of BM precursors, observed in the vast majority of PS patient BMAs, is not influenced by the patient's age at sampling. Ring sideroblasts, the other feature of PS BMAs, are less frequent than progenitor vacuolization but increase significantly after 6 months of age. These abnormalities are just as common in patients with or without hematological symptoms, suggesting that BMA should be performed in all suspected PS cases, despite the absence of anemia. PS is a multisystem disorder requiring early diagnosis and a coordinate multidisciplinary management, involving clinicians and clinical biologists.

## 1. Introduction

Pearson syndrome (PS) is a rare mitochondrial oxidative phosphorylation disorder caused by a single large-scale mitochondrial DNA (mtDNA) deletion (SLSMD) [1, 2]. The prevalence of PS is estimated at 1 in 1,000,000, with nearly 200 cases reported worldwide [3–6]. Initial symptoms typically appear during the neonatal period or in early infancy and include severe macrocytic or normocytic hyporegenerative anemia, frequently accompanied by neutropenia (80%) and/or thrombocytopenia (72%) [3, 4, 6]. Hematological signs are rapidly followed by progressive symptoms including failure to thrive, pancreatic insufficiency, muscular hypotonia, kidney insufficiency, endocrinopathies, and lactic acidosis [2–8]. Currently, therapeutic approaches for PS patients are only symptomatic, and over 50% die before the age of 6 years [3–6]. However, up to two-thirds of PS patients show a spontaneous resolution of anemia during their clinical course, though this resolution seems to have little impact on the prognosis [6]. Patients surviving into late infancy often experience a phenotypic evolution to Kearn–Sayre syndrome (KSS) characterized by progressive external ophthalmoplegia, pigmentary retinal degeneration, cerebellar ataxia, and cardiac conduction block [3, 4, 9]. Bone marrow (BM) aspirates in PS patients are characterized by vacuolization of myeloid and erythroid precursors, frequently accompanied by ring sideroblasts (70%–85%) and dysplasia in all lineages [3, 6, 10]. However, the timing of these BM anomalies remains unclear. Here, we report three new cases of PS diagnosed in our institution and review the literature to summarize the clinical and hematological features of PS.

### 1.1. Case Report 1

**Figure 1:**
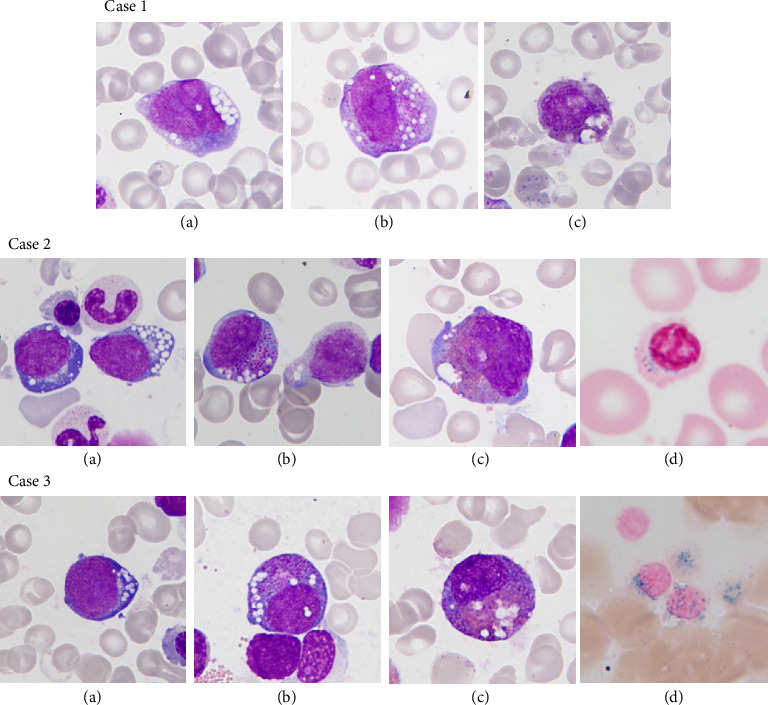
Bone marrow aspiration of the three presented cases. (a–c) May–Grünwald Giemsa coloration shows a vacuolization in erythroid and myeloid progenitors. Original magnification × 1000. (d) Perls' Prussian blue iron stain shows the presence of ring sideroblasts. Original magnification × 1000.

The first patient is a full-term girl, born to an in vitro fertilization through sperm donor to a Belgian mother with an unremarkable medical history. The pregnancy and delivery were uncomplicated, and the birth weight was 3200 g. At 7 months old, the girl was admitted to the pediatric intensive care unit (ICU) due to severe lactic acidosis in the setting of flu infection. At admission, she was apathic, with fluctuating level of consciousness, Kussmaul respiration, pallor, tachycardia, myosis, and hepatomegaly. Measurements showed a weight of 6200 g and a height of 68.2 cm. Laboratory findings revealed severe hyporegenerative anemia, hypoglycemia, severe metabolic acidosis, and hyperlactatemia (Table 1). Metabolic workup indicated generalized organic aciduria and increased plasma amino acids taurine, proline, and histidine. During the first week at ICU, she developed persistent moderate thrombocytopenia (min: 30 × 10^6^ L) and proximal tubular dysfunction with significant electrolyte imbalance. The BMA performed to investigate the bicytopenia revealed erythropoiesis dysplasia (46%), dysgranulopoiesis, vacuolization of myeloid and erythroid precursors (7%), and ring sideroblasts (20%) (Figure 1). Due to the child's young age and low diagnostic added value, trephine biopsy was not performed. Due to BM anomalies, persistent hyperlactatemia, and renal tubulopathy, PS was suspected. mtDNA sequencing revealed the presence of a heteroplasmic (NC_012920.1): m8483-13459 SLSMD, consistent with PS.

Since her initial hospitalization, the patient developed persistent severe anemia and became dependent on monthly red blood cell (RBC) transfusions. She also exhibited persistent hyperlactatemia and exocrine pancreatic insufficiency. Her proximal tubular dysfunction progressively deteriorated, requiring increasing intravenous electrolyte supplementation and ultimately leading to the initiation of parenteral nutrition (PN) at 21 months of age. Starting with the placement of the PN, electrolyte administration led to a sudden weight gain (+2SD), which had been stable previously. At 21 months, a gradual delay in motor development also appeared, mainly marked by axial hypotonia and treated with Bobath physiotherapy. From the age of 22 months, the patient experienced several episodes of pancreatitis, accompanied by diabetes flare-ups managed with subcutaneous insulin. Central respiratory failure developed as well, requiring overnight bilevel positive airway pressure non-invasive ventilation. Around 28 months of age, the refractory anemia deteriorated, and the frequency of RBC transfusion increased to every 2–3 weeks. The patient experienced multiple hospitalizations for viral infections and invasive medical device infections. Each infection was associated with metabolic decompensation, worsening renal tubulopathy, hyperlactatemia, respiratory failure, and axial hypotonia. The patient died at 39 months due to respiratory failure caused by progressive encephalopathy.

### 1.2. Case Report 2

The second patient is a full-term girl, born with 2450 g after an intrauterine growth restriction and delivered via cesarean due to a bicornuate uterus. She is the second child of a non-consanguineous Caucasian couple, without any relevant medical history. On the 10th day of life, a blood count revealed severe hyporegenerative anemia and severe neutropenia (Table 1). At 2 months old, hematologic and metabolic workup revealed persistent thrombocytopenia, significant fumaric aciduria, and slightly increased plasma amino acids alanine and glutamine. BM aspiration revealed a normal maturation of the three cell lines but with vacuolization of myeloid and erythroid precursors and ring sideroblasts (15%) (Figure 1). Due to the child's young age and low diagnostic added value, trephine biopsy was not performed. Acquired causes of sideroblastic anemia were ruled out based on normal copper levels, the absence of myelodysplastic syndrome on BMA, and the absence of toxic or drug intake. A genetic panel was also carried out and came back negative (no mutations in the SCL19A2, SLC25A19 genes TRK1). In addition to monthly RBC transfusions and occasional platelet transfusions, the patient received thiamine and riboflavin supplements until mtDNA results were available. However, these supplements did not improve her condition. mtDNA sequencing shows the presence of a heteroplasmic (NC_012920.1): m8483-13459 SLSMD, consistent with PS.

From 12 months of age, the patient showed progressive failure to thrive (weight (−2SD) and height (−2SD) decrease). Moreover, due to weight loss and feeding difficulties, enteral feeding was initiated at 14 months, followed by endoscopic gastrostomy 1 year later. Around 18 months of age, the frequency of RBC transfusion increased to every 2–3 weeks, although the neutropenia became mild and intermittent. Proximal tubular dysfunction developed at 20 months of age, causing significant electrolyte imbalances and requiring increased supplementation. The patient suffered multiple hospitalizations for recurrent infections, including several invasive infections related to medical devices. Following the development of tubulopathy, each infection led to metabolic decompensation aggravating lactic acidosis, electrolyte imbalances, and sometimes required PN. Two episodes of viral infections were also accompanied by rapidly resolving acute pancreatitis, without deterioration of endocrine or exocrine function. However, around 26 months, she suffered from two episodes of tetany due to severe hypocalcemia and was subsequently treated with continuous intravenous calcium supplementation. The patient died at 30 months due to cardiorespiratory failure following a central line removal surgery.

### 1.3. Case Report 3

The third case is a full-term boy, born after an uncomplicated pregnancy and delivery, with a birth weight of 3285 g. He is the second child of a non-consanguineous Portuguese couple, with unremarkable medical history. At 8 months of age, he was admitted to the emergency room for fever, asthenia, anorexia, cough, and diarrhea. Measurements showed a weight of 7490 g and a height of 67.5 cm, and physical examination was unremarkable except an erythematous pharynx and slight weight loss. On admission, full blood count revealed pancytopenia with severe hyporegenerative anemia, thrombopenia, and severe neutropenia (Table 1). The blood smear revealed 81% of lymphocytes but no morphologic signs of malignant hemopathy. Other laboratory findings were unremarkable except for positive PCR and cultures for influenza virus. At 9 months of age, BMA revealed a normal maturation of all three cell lines but showed a slight excess of blasts (8.5%), striking vacuolization of myeloid and erythroid precursors (7%), and ring sideroblasts (53%) (Figure 1). Immunophenotypic analysis revealed no lymphoid lineage anomalies and identified blasts as hematogones CD19^+^ CD34^+/−^ CD10^+^ CD38^+^ (10.9%) and myeloblasts CD34^+^ CD177^+^ CD13^+^ CD33^+^ (2.5%). Due to the child's young age and low diagnostic added value, trephine biopsy was not performed. Two months later, a second BMA performed at a distance from the viral infection produced similar results. Metabolic assessment showed a significantly increased plasma amino acid alanine. mtDNA analysis results showed the presence of a heteroplasmic (NC_012920.1): m8483-13459 SLSMD, consistent with PS. Since his initial admission, the patient developed persistent pancytopenia and neutropenia (Min: PLT: 84 × 10^9^ L; PMN: 0.17 × 10^3^ L) and became dependent on monthly RBC transfusions until he reached the age of 12 months. The patient is now 18 months old and shows no signs of neurodevelopmental disorders or endocrine disorders so far. However, he has been treated for pancreatic insufficiency since 15 months old and he is followed for slight proximal tubular dysfunction.

## 2. Materials and Methods for the Review of the Literature

**Figure 2:**
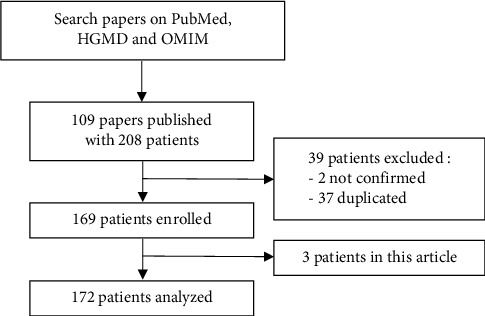
Case-screening flowchart.

A PubMed search was performed for relevant studies published between 1979 and 2024 using the terms “Pearson,” “Pearson's syndrome,” “Pearson marrow-pancreas syndrome,” and “mtDNA deletion syndrome” (Figure 2). Only PS cases confirmed by DNA analysis were included in this review. After removing duplicates, 169 PS cases from 75 published reports were considered. The age of onset was defined as the time when symptoms first appeared. Initial symptoms were defined as those observed at first presentation. The overall survival was calculated to the date of death from any cause or the date of the last follow-up. Survival analysis was performed by the Kaplan–Meier analysis.

### 2.1. Age of Onset and First Symptoms

**Figure 3:**
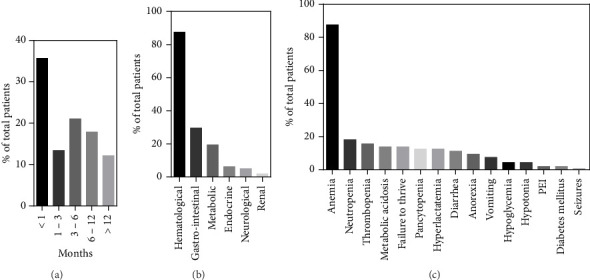
Onset characteristics of PS patients. (a) Distribution of the age of onset in 157 patients. (b) The percentage of affected systems at onset. (c) The percentage of symptoms observed at onset (PEI, pancreatic exocrine insufficiency).

The age of the first symptoms was specified for 157/172 patients, including our index cases. This mainly occurs in the first month of life (56/157, 35.6%), with a significant decrease from the second month onwards (Figure 3). The median onset age was 3 months (interquartile range (IQR): 6 months) with a minimum at birth and a maximum at 36 months. The most common initial symptom was hyporegenerative anemia, observed in 87.5% (140/160) of cases and the only symptom in 31.3% (50/160) of them. Anemia could be accompanied by other hematological alterations such as thrombocytopenia (25/160, 15.6%), neutropenia (29/160, 18.1%), or pancytopenia (27/160, 16.9%). Additionally, patients presented a wide spectrum of non-hematological symptoms: 29.6% (40/160) showed gastrointestinal symptoms with failure to thrive, vomiting, diarrhea, anorexia, and exocrine pancreatic insufficiency; 19.4% (31/160) metabolic disorders such as metabolic acidosis and hyperlactatemia; 6.2% (10/160) endocrine disorders with hypoglycemia and diabetes mellitus; 5% (8/160) neurological disorders with seizures and hypotonia; 1.2% (2/160) of renal disorders with renal tubulopathy. Among patients without any hematological abnormalities (12.5%, 20/160), growth retardation was the most common symptom (55%, 11/20), followed by anorexia, diarrhea, vomiting (25%, 5/20), and metabolic acidosis (20%, 4/20).

### 2.2. Morphological Characteristics of BM Smears

**Figure 4:**
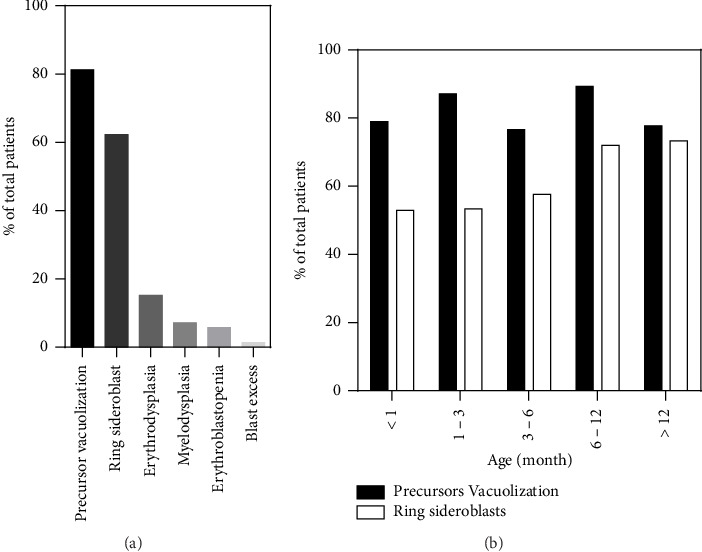
BMA features of PS patients. (a) Distribution of BM abnormalities across 138 patients. (b) BM abnormalities in correlation with the time of completion of the BMA.

A BM aspiration was performed in 138/172 cases, of which 126/138 clearly indicated the age at sampling and 122/138 smears were additionally stained with a Perls coloration. The most frequently observed abnormality was the vacuolization of hematopoietic precursors, present in 81.2% (112/138) of cases, followed by the presence of ring sideroblasts at the Perls in 62.3% of cases (76/122) (Figure 4). In addition to these common characteristics, 18.8% (26/138) of BM presented other abnormalities such as erythrodysplasia (21/138, 15.2%), more global myelodysplasia (10/138, 7.2%), erythroblastopenia (8/138, 5.7%), and blast excess (2/138, 1.4%). Hypocellular or aplasic BMAs were observed in 31.1% (43/138) of cases, with 74% (32/43) showing precursor vacuolization. The timing of the first BM aspiration varied, with 15.1% (19/126) performed within the first month of life, 24.6% (31/126) between 1 and 3 months, 23.8% (30/126) between 3 and 6 months, 22.2% (28/126) between 6 and 12 months, and 14.3% (18/126) after 12 months. Precursor's vacuolization was consistently observed across all age groups, with the highest prevalence at 6–12 months (89.3%, 25/28) and the lowest at 3–6 months (76.1%, 23/30). Ring sideroblasts were increasingly observed with age, from 52.9% (9/17) in those less than 1 month old to 73.3% (11/15) in those older than 12 months. The majority of hypocellular BMAs showing none of PS morphological characteristics were harvested between 3 and 6 months of age (5/11). The remainder were evenly distributed over the other time intervals.

### 2.3. Clinical Features Observed During the Course of the Disease

**Figure 5:**
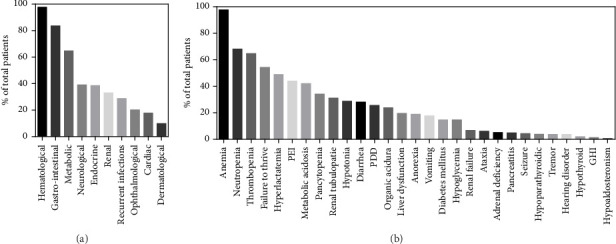
Clinical evolution of PS patients. (a) The percentage of systems affected during the course of the disease in 163 patients. (b) The percentage of symptoms observed during the course of the disease (PEI, pancreatic exocrine insufficiency; PDD, psychomotor developmental delay; GHI, growth hormone insufficiency).

The patient's clinical evolution during the course of the disease was described for 167/172 cases. While hyporegenerative anemia was the most common initial symptom—as described previously—it is also the one that manifested itself predominately as the disease progresses (97.6%, 160/164). Hematologic abnormalities also included neutropenia (68%, 102/150), thrombocytopenia (64.6%, 106/164), and pancytopenia (34.4%, 56/164) (Figure 5). Gastrointestinal symptoms (83.5%, 137/164) were the second most common features, with failure to thrive (54.3%, 89/164), exocrine pancreatic insufficiency (43.9%, 72/164), diarrhea (28%, 46/164), liver dysfunction (19.5, 32/164), anorexia (18.9%, 31/164), vomiting (17.7%, 29/164), and pancreatitis (4.9%, 8/164). Metabolic disorders (64.6%, 106/164) included hyperlactatemia (48.8%, 80/164), metabolic acidosis (42%, 69/164), and abnormal organic aciduria (23.8%, 39/164). Neurological symptoms (39%, 64/164) consisted of hypotonia (28.7%, 43/150), psychomotor development delay (25.6%, 42/164), ataxia (6.1%, 10/164), seizure (4.3%, 7/164), tremors (3.6%, 6/164), and hearing disorders (3.6%, 6/164). Endocrine disorders (37.8%, 62/164) included diabetes mellitus (14.6%, 24/164), hypoglycemia (14.6%, 24/164), adrenal deficiencies (5.1%, 8/157), hypoparathyroidism (3.8%, 6/157), hypothyroidism (1.9%, 3/157), and growth hormone deficiencies (1.2%, 2/157). Renal symptoms (32.9%, 54/164) were renal tubulopathy (31.1%, 53/164) and renal failure (6.7%, 11/164). Finally, 28.6% (47/164) of patients experienced recurrent infections, 20.1% (33/164) ophthalmological disorders, 17.7% (29/164) heart disorders, and 9.7% (16/164) dermatological disorders. Finally, 39.2% (20/51) of patients surviving beyond 5 years were diagnosed with KSS.

### 2.4. Survival and Cause of Death

**Figure 6:**
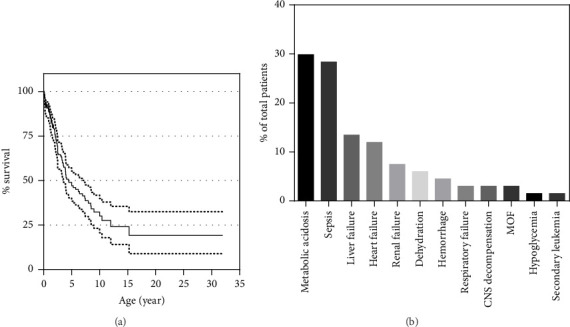
Prognosis of PS patients. (a) Kaplan–Meier curves for the overall survival probability of 172 patients. (b) Causes of death distribution across the 91 death patients (MOF, multiple organ failure).

The 3- and 5-year overall survival of the cohort were, respectively, 64.5% (95% confidential interval 56.4%–71.6%) and 47.7% (95% confidential interval 39%–55.9%), with a plateau at 19.5% (95% confidential interval 9%–32.8%) (Figure 6). Among the 172 patients, 90 had died at a median age of 54 months (range: 0.5–383 months) and 82 were alive at the last follow-up (median age of 48 months (range: 3–252)). The cause of death was specified for only 67 of the 90 patients, the most frequent being metabolic acidosis (20/67, 29.9%), closely followed by sepsis (19/67, 28.4%). Other causes, in the order of frequency, were hepatic failure (9/67, 13.4%), cardiac failure or arrest (8/67, 11.9%), renal failure or tubulopathy (5/67, 7.5%), dehydration (4/67, 6%), hemorrhage (3/67, 4.5%), respiratory failure (2/67, 3%), and multiple organ failure (2/67, 3%).

## 3. Discussion and Summary

PS was first described by Pearson et al. in 1979 as a rare and often fatal disease of early childhood, characterized by refractory sideroblastic anemia with BM precursors vacuolization and pancreatic fibrosis [2]. Since its initial description, PS has been recognized as a multisystem disorder caused by single large-scale mtDNA deletions [1]. The proportion of wild-type and mutated mtDNA varies across different tissues, called heteroplasmy, likely due to random distribution during early cell division [11]. Cellular dysfunction appears only if the level of heteroplasmy exceeds a certain threshold, impairing the oxidative phosphorylation chain and ultimately increasing anaerobic metabolism [11–13]. PS-associated deletions are mostly sporadic and exhibit significant diversity, with the most common being a 4977-bp deletion found in 22.2% of reported cases [14]. This diversity in deletion sizes and levels of heteroplasmy may contribute to the phenotypic variability observed in PS. However, the correlation between genotype and phenotype remains controversial, and the level of heteroplasmy has not been significantly correlated with the age of onset or the clinical course of the disease [4, 14]. Among our three cases, none of the families had a history of genetic disease or symptoms indicative of mitochondrial disease and all three patients exhibited the most common 4977-bp SLSMD.

The age of onset is generally early, with some documented cases even beginning in utero, presenting as anemic hydrops fetalis or prenatal pancytopenia [7, 8, 15]. Our data support the early onset trend with 35.6% of patients developing their first symptoms within the first month of life, which is quite comparable with other studies [7, 14]. The onset of symptoms occurs across a wide age range, up to 36 months, reflecting the clinical variability of PS. This variability is illustrated by the different ages of presentation in our three index cases (2, 7, and 8 months at onset). Early symptoms of PS may include severe hyporegenerative anemia, present in 87.5% of cases in our study, which closely matches Ying et al. results [6, 14]. This consistency between studies confirms that anemia is the main symptom of PS at presentation. Other hematological abnormalities may accompany anemia early on, such as thrombocytopenia and/or neutropenia, as shown in the literature [3, 6, 14]. Interestingly, none of the reviewed cases showed hematological abnormalities without hyporegenerative anemia, suggesting that PS should not be considered for these patients. Anemia as the only symptom at presentation was observed in 31.3% of our cases, which corroborates the findings of Manea et al. on newborns but is lower than those of Yoshimi et al. and Ying et al. [7, 11, 14]. However, these variations may be due to selection bias in onset symptoms. The presence of anemia without other symptoms could lead to misdiagnosis of Diamond–Blackfan anemia and congenital sideroblastic anemia [16–18]. Therefore, PS should be excluded in all infants with hyporegenerative anemia of unknown etiology up to at least 3 years. Beyond hematological abnormalities, PS patients present a whole series of non-hematological symptoms from onset. Gastrointestinal and metabolic disorders (29.6% and 19.4%) were the most common, with failure to thrive and metabolic acidosis/hyperlactatemia being the most common symptoms. These observations are quite comparable to those reported by Ying et al., though the exact composition of symptom groups varies between the two studies [14]. These results suggest that PS should also be excluded in all children presenting with hyporegenerative anemia accompanied by failure to thrive or metabolic acidosis. The results of the review are well illustrated by our three index cases, each presenting hyporegenerative anemia at the beginning, associated with hyperlactatemia for two of them and with gastrointestinal disorders for the other. Furthermore, failure to thrive and metabolic acidosis are among the most frequent symptoms in patients without hematological disorders, suggesting that PS should be considered even in patients presenting lactic acidosis or failure to thrive despite the absence of anemia.

The diagnosis of PS is often difficult due to the clinical variability and the absence of specific symptoms. However, BM anomalies, particularly vacuolization of erythroid and myeloid progenitors, are highly suggestive of PS [3, 6, 10]. In our review, up to 81.2% of patients' BM showed precursor vacuolization, although these proportions slightly vary from study to study [3, 6, 10, 19]. These variations may be due to the small cohort in the studies. It is important to note that precursor vacuolization in the pediatric population is not exclusive to mitochondrial disorders and can be seen in various other conditions, such as myelodysplastic syndromes, protein–calorie malnutrition (e.g., marasmus, kwashiorkor), copper deficiency (e.g., zinc poisoning, penicillamine), and drug toxicity (e.g., chloramphenicol, linezolid) [20]. Ring sideroblasts are the other typical feature of PS BM (62.3%), although they show great variability between different studies and are less frequent than progenitor vacuolization [3, 6, 10, 19]. In fact, they can also be observed in myelodysplastic syndromes, copper deficiency, drug toxicity (e.g., chloramphenicol, linezolid, isoniazid), and other congenital sideroblastic anemias such as thiamine-responsive sideroblastic anemia, congenital anomalies of folate, or cobalamin metabolism [21]. Additionally, the PS BM may show dysplasia in myeloid and/or erythroid lineages, consistent with previous reports [3, 6, 10]. However, the proportion of ring sideroblasts and lineage dysplasia may be under- or over-estimated, as not all reports comment on these findings. Interestingly, BM abnormalities are just as common in patients with or without hematological symptoms, suggesting that a BM aspiration should be performed in all patients with a suspected PS, despite the absence of anemia. Although trephine biopsy is the gold standard to assess BM cellularity, hypocellular BMAs were reviewed to evaluate the impact of poor marrow harvesting. Morphological features of PS appear in similar proportions in BMAs from the overall population and in hypocellular/aplasic BMAs, suggesting minimal bias due to poor sampling. In our three cases, each patient's BM showed precursor vacuolization and ring sideroblasts. However, erythrodysplasia and myelodysplasia were present only in Case 1. Tadiotto et al. reported that BM aspirations performed during the first month of life are less informative than those performed later, mainly due to a lower presence of vacuolated precursors and ring sideroblasts [10]. This trend was not observed in our patients, nor was there a clear evolutionary pattern of precursor vacuolization depending on the age at sampling. In contrast, the frequency of ring sideroblasts increased significantly from 6 months. These variations can be explained by the large difference in the number of cases studied. However, a slight underestimation of the morphological characteristics present in BMAs sampled between 3 and 6 months is possible due to poor sampling bias.

To date, therapeutic approaches for PS patients are only symptomatic, contributing to the high mortality associated with this disease. Our review confirms the poor prognosis with 52.3% mortality at a median age of 54 months. Those findings are consistent with the recent literature, while historical studies reported much higher mortality rates [3, 6, 14]. Variations between historical and recent studies can probably be explained by the general improvement in supportive care: RBC transfusions for anemia, bicarbonate administration to control metabolic acidosis, and GCSF to fight infection in neutropenia [4]. Interestingly, patients with hematological recovery show a similar survival, as they develop various severe non-hematological complications as well [6]. There are currently no predictive factors for the survival of PS patients. Most patients died due to metabolic decompensation, mainly due to infections or sepsis, consistent with literature reports. Interestingly, we showed that 39.2% of patients surviving beyond 5 years were diagnosed with KSS, which closely matches Farruggia et al. (50%, 3/6) results [3]. However, these results may be underestimated due to a significant diagnostic bias. Out of our three index cases, two died at 30 and 39 months, respectively, due to cardiorespiratory failure and respiratory failure.

## Figures and Tables

**Table 1:** Laboratory data at presentation of the three reported cases.

	Case 1	Case 2	Case 3	Reference range	Units
Hb	58	51	50	9–14	(g/L)
MCV	94	106	104	77–115	(fL)
PLT	308	159	95	150–440	(10^6^/L)
WBC	9.4	3.2	5.6	5–18	(10^3^/L)
PMN	4.6	0.46	0.7	1–9	(10^3^/L)
LYMPH	4.3	2.5	4.5	2.5–15	(10^3^/L)
ARet	20	47.7	15.4	22.5–147	(10^3^/L)
CRP	55.4	< 1	9.8	< 5	(mg/L)
Folate		> 20	16.7	> 4.6	(μg/L)
Vit B12		382	677	197–771	(ng/L)
Lactatemia	10		3.5	< 4.1	(mmol/L)
Glycemia	45		90	70–100	(mg/L)

Abbreviations: ARet, absolute reticulocytes; CRP, C-reactive protein; Hb, hemoglobin; LYMPH, lymphocyte; MCV, mean corpuscular volume; PLT, platelet; PMN, polymorphonuclear leukocyte; Vit B12, vitamin B12; WBC, white blood cell.

## Data Availability

The data that support the findings of this study are available from the corresponding author upon reasonable request.

## Nomenclature

PS: Pearson syndrome
mtDNA: Mitochondrial DNA
BMA: Bone marrow aspiration
KSS: Kearn–Sayre syndrome
ICU: Intensive care unit
IQR: Interquartile range
SLSMD: Single large-scale mitochondrial DNA deletion
RBCs: Red blood cells
PN: Parenteral nutrition
PCR: Polymerase chain reaction

## Supporting Information

Table S1: Review of the literature, onset ages, and prognosis.
