# Supplementary material for: Clinical and Morphological Bone Marrow Characteristics of Pearson Syndrome: About Three Consecutive Cases and Review of the Literature
Source: Case Rep Pediatr. 2025 May 18;2025:3076141. doi: 10.1155/crpe/3076141 (PMC12103953; doi:10.1155/crpe/3076141)
Supplement: Supporting Information — Additional supporting information can be found online in the Supporting Information section. [file 3076141.f1.docx]

**Supplemental table S1: Review of literature, onset ages and prognosis.**

| Author | Case N° | Sex | onset age | Prognosis |
| --- | --- | --- | --- | --- |
| (Pearson et al., 1979) | 1 | female | at birth | died at 2 years, 2 months |
|  | 2 | male | 14 months | died at 2 years, 5 months |
|  | 3 | female | 3 weeks | alive at 3 years, 6 months |
|  | 4 | male | 1 month | alive at 3 years |
| (Stoddard et al., 1981) |  | female | at birth | died at 1 month, 24 days |
| (Bernes SM. et al., 1993) |  | male | 13 months | alive at 4 years |
| (Rötig et al., 1990) | 1 | male | 2 months | died at 14 months |
|  | 2 | female | 1 months | died at 2 years, 6 months |
|  | 3 | female | at birth | died at 2 years, 1 months |
|  | 4 | female | 7 months | died at 2 years, 6 months |
|  | 5 | male | 9 months | died at 2 years |
| (McShane MA et al., 1991) |  | male | 8 days | alive at 8 years |
| (Baerlocher et al., 1992) |  | male | at birth | alive at 7 years, 2 months |
| (de Vries et al., 1992) |  | female | at birth | died at 16 months |
| (Gibson et al., 1992) | 1 | male | 9 months | died at 2 years |
|  | 2 | female | 5 months | alive at 7 months |
|  | 3 | female | 1 year | died at 3 years, 6 months |
|  | 4 | female | 1 month | alive at 10 years |
| (Giirgeya et al., 1992) | 1 | female | 5 months | died at 19 months |
|  | 2 | male | 2 months | died at 13 months |
| (Lichter-Konecki et al., 1992) |  | female | 1 week | died at 3 years, 7 months |
| (Morikawa et al., 1993) |  | male | 1 month | died at 2 years, 2 months |
| (Ribes et al., 1993) |  | male | 16 months | alive at 3 years |
| (Superti-Furga et al., 1993) |  | female | at birth | died at 19 months |
| (Kapsa et al., 1994) |  | male | 7 months | died at 3 years, 4 months |
| (Niaudetl et al., 1994) |  | female | 6 months | alive at 7 years, 8 months |
| (Rötig et al., 1995) | 2 | male | at birth | died at 2 years |
|  | 4 | female | 5 weeks | died at 3 years, 6 months |
|  | 5 | female | at birth | alive at 9 years |
|  | 6 | female | 7 months | died at 2 years, 6 months |
|  | 7 | female | 5 months | alive at 7 years |
|  | 10 | female | at birth | died at 3 months |
|  | 11 | male | 4 weeks | alive at 17 years |
|  | 12 | female | 7 months | died at 2 years, 6 months |
|  | 14 | female | 4 months | died at 3 years, 10 months |
|  | 15 | female | 3 years | alive at 11 years |
|  | 18 | male | 9 months | alive at 2 years |
|  | 19 | male | NA | alive at 3 years |
|  | 20 | male | 1 month | died at 2 months |
|  | 21 | male | 4 months | alive at 13 years |
| (Smith et al., 1995) |  | female | at birth | died at 5 years |
| (Giirgey et al., 1996) |  | male | 2 days | died at 41 days |
| (Santorelli et al., 1996) |  | male | 7 months | died at 4 years |
| (Kleinle S. et al., 1997) |  | male | 2 days | died at 3 months |
| (Muraki K. et al., 1997) |  | female | at birth | died at 14 days |
| (Muraki K et al., 1997) | 1 | female | 18 days | alive at 17 years |
|  | 2 | male | 3 days | alive at 5 years |
| (Seneca et al., 1997) |  | male | 5 months | alive at 5 years |
| (Cursiefen C. et al., 1998) |  | female | 7 months | alive at 6 years |
| (Toth T et al., 1998) |  | male | NA | died at 2 years, 4 months |
| (Krahenbtihl et al., 1999) |  | male | 2 days | died at 3 months |
| (Lacbawan et al., 2000) |  | female | 16 months | alive at 8 years |
| (Krauch et al., 2002) |  | female | 3 months | alive at 5 years, 5 months |
| (Shanske et al., 2002) |  | male | 4 months | died at 1 years |
| (Knerr et al., 2003) |  | male | 5 months | alive at 2 years |
| (Li C. H. et al., 2003) |  | female | at birth | alive at 15 months |
| (Jacobs et al., 2004) | 1 | female | 16 months | died at 3 years, 3 months |
|  | 2 | female | 1 year | died at 10 years |
| (Lohi et al., 2005) |  | female | at birth | alive at 9 months |
| (Giese et al., 2007) |  | male | at birth | died at 11 weeks |
| (Kefala-Agoropoulou et al., 2007) |  | female | 2 days | died at 13 months |
| (Lee et al., 2007) |  | female | 16 days | died at 3 years, 4 months |
| (Maguluri & Recchia, 2007) |  | male | NA | alive at 32 years |
| (Topaloğlu et al., 2008) | 1 | male | 7 months | died at 7 months |
|  | 2 | female | 2 months | died at 4 years, 6 months |
| (Atale et al., 2009) |  | male | 4 months | alive at 4 years |
| (Manea et al., 2009) | 1 | male | at birth | alive at 1 years, 6 months |
|  | 2 | male | at birth | alive at 4 months |
| (Momont AC & Trobe JD, 2009) |  | female | 1 year | alive at 3 years, 9 months |
| (Morel et al., 2009) |  | male | at birth | died at 18 months |
| (Arzanian et al., 2010) |  | female | 4 months | alive at 6 months |
| (Köklü et al., 2010) |  | female | NA | alive at 21 years |
| (Ayed et al., 2011) |  | female | 2 months | died at 21 months |
| (Tumino et al., 2011) | 1 | male | 9 months | died at 8 years |
|  | 2 | female | 4 months | died at 8 years, 6 months |
|  | 3 | male | 1 month | died at 4 months |
|  | 4 | male | 4 months | died at 6 months |
| (Binder et al., 2012) |  | male | 8 months | alive at 9 months |
| (Williams et al., 2012) | 1 | female | at birth | died at 2 years |
|  | 2 | male | 2 years | alive at 5 years |
| (Kasbekar et al., 2013) |  | male | 3 months | died at 12 years |
| (Baertling et al., 2014) |  | female | 6 months | alive at 2 years |
| (Broomfield et al., 2015) | 1 | female | at birth | died at 5 years, 3 months |
|  | 2 | male | at birth | died at 7 years, 5 months |
|  | 3 | male | at birth | alive at 8 years, 6 months |
|  | 4 | female | at birth | died at 2 years, 6 months |
|  | 5 | female | at birth | died at 4 months |
|  | 6 | male | at birth | alive at 6 years |
|  | 7 | female | at birth | died at 14 months |
|  | 8 | male | 2 months | died at 2 years, 4 months |
|  | 9 | female | 5 months | died at 4 years, 5 months |
|  | 10 | male | 16 months | alive at 6 years |
|  | 11 | male | 4 months | alive at 2 years, 5 months |
| (Gagne et al., 2014) | 1 | male | at birth | died at 2 years, 5 months |
|  | 2 | NA | NA | alive at 3 years |
|  | 3 | male | at birth | alive at 7 years |
|  | 4 | male | at birth | died at 2 years, 1 month |
|  | 5 | NA | NA | alive at 9 years |
|  | 6 | male | at birth | alive at 1 year |
|  | 7 | female | 3 months | died at 19 months |
|  | 8 | female | at birth | alive at 6 years |
| (Crippa et al., 2015) | 1 | female | 2 months | died at 2 years, 1 month |
|  | 2 | male | 6 months | alive at 4 years |
|  | 3 | female | 6 months | alive at 3 years, 6 months |
|  | 4 | female | 6 months | alive at 2 years |
| (Farruggia et al., 2016) | 1 | NA | NA | died at 6 years, 5 months |
|  | 2 |  |  | alive at 2 years, 11 months |
|  | 3 |  |  | alive at 3 years, 8 months |
|  | 4 |  |  | died at 5 years, 8 months |
|  | 5 |  |  | alive at 6 years, 7 months |
|  | 10 |  |  | died at 10 years, 5 months |
|  | 11 |  |  | died at 3 years, 11 months |
| (Park et al., 2015) |  | female | 4 months | alive at 2 years, 1 month |
| (Sato et al., 2015) |  | female | at birth | died at 3 years, 11 months |
| (Chen et al., 2016) |  | male | at birth | alive at 2 y |
| (Hiebert et al., 2016) |  | male | 6 months | died at 3 years, 2 months |
| (Falcon & Howard, 2017) |  | male | 3 months | alive at 3 months |
| (Tadiotto et al., 2018) |  | female | 3 days | alive at 3 years, 6 months |
| (Khasawneh et al., 2018) |  | male | 3 months | died at 3 months |
| (Tesarova et al., 2019) | 1 | male | 1day | died at 11 months |
|  | 2 | female | 1day | died at 16 months |
|  | 3 | female | 3 months | alive at 4 years |
|  | 4 | female | 3 months | alive at 5 years |
|  | 5 | male | 18 months | alive at 11 years |
| (Akesson et al., 2019) | 1 | female | 4 days | died at 11 weeks |
|  | 2 | male | 4 hours | died at 16 weeks |
| (Mats & Cortez, 2020) |  | female | NA | alive at 7 years |
| (Pronman et al., 2019) |  | male | 9 months | alive at 15 months |
| (Reddy et al., 2019) |  | male | 1 month | died at 3 months |
| (Wild et al., 2020) | 1 | male | 2 years, 1 month | alive at 4 years |
|  | 2 | female | 2 years | alive at 9 years |
|  | 3 | female | 18 months | alive at 5 years |
|  | 4 | female | 21 months | alive at 2 years |
|  | 5 | male | at birth | died at 10 months |
| (Liu et al., 2021) |  | male | 18h | alive at 13 months |
| (Nilay & Phadke, 2020) |  | female | 6 months | alive at 5 years |
| (Yoshimi et al., 2022) | 1 | male | 4 months | died at 3 years, 11 months |
|  | 2 | male | 5 months | died at 8 years, 5 months |
|  | 3 | female | at birth | died at 15 years, 3 months |
|  | 4 | female | 5 months | died at 3 years |
|  | 5 | male | at birth | died at 6 years, 2 months |
|  | 6 | male | 5 months | died at 9 years |
|  | 7 | male | at birth | died at 4 years, 1 month |
|  | 8 | female | 6 months | died at 7 years, 4 months |
|  | 9 | male | 5 months | died at 3 years, 11 months |
|  | 10 | female | 2 years, 1 month | alive at 1 years, 10 months |
|  | 11 | female | 2 years, 7 months | alive at 9 years, 11 months |
|  | 12 | male | 8 months | alive at 16 months |
|  | 13 | male | 5 months | alive at 7 years, 6 months |
|  | 14 | male | 6 months | alive at 5 years |
|  | 15 | female | 8 months | alive at 4 years, 5 months |
|  | 16 | male | at birth | died at 2 years, 3 months |
|  | 17 | male | 5 months | died at 6 years, 11 months |
|  | 18 | female | 3 months | alive at 21 months |
|  | 19 | female | 5 months | alive at 7 months |
|  | 20 | male | 8 months | died at 14 months |
|  | 21 | female | 12 months | died at 17 months |
|  | 22 | male | 4 months | alive at 2 years, 8 months |
|  | 23 | female | 1 month | died at 4 years |
|  | 24 | male | at birth | alive at 12 months |
|  | 25 | female | at birth | alive at 16 months |
| (Son et al., 2022) | 1 | male | 2 months | died at 11 months |
|  | 2 | female | 2 years | alive at 12 years |
|  | 3 | male | 5 months | died at 5 years |
|  | 4 | female | 1 months | alive at 3 years |
| (Tedjaseputra et al., 2023) |  | male | 4 weeks | alive at 5 months |
| (Ying et al., 2022) |  | male | 7 months | died at 2 years, 7 months |
| (Belgacem et al., 2023) |  | male | at birth | alive at 7 years, 6 months |
| (Shoeleh et al., 2023) |  | male | 2 months, 3weeks | alive at 3 months |
| This study | 1 | female | 7 months | died at 39 months |
|  | 2 | female | 2 months | alive at 30 months |
|  | 3 | male | 8 months | alive at 18 months |

**Bibliography**

Akesson, L. S., Eggers, S., Love, C. J., Chong, B., Krzesinski, E. I., Brown, N. J., Tan, T. Y., Richmond, C. M., Thorburn, D. R., Christodoulou, J., Hunter, M. F., Lunke, S., & Stark, Z. (2019). Early diagnosis of Pearson syndrome in neonatal intensive care following rapid mitochondrial genome sequencing in tandem with exome sequencing. *European Journal of Human Genetics*, *27*(12), 1821–1826. https://doi.org/10.1038/s41431-019-0477-3

Arzanian, M. T., Eghbali, A., Karimzade, P., Ahmadi, M., Houshmand, M., & Rezaei, N. (2010). mtDNA Deletion in an Iranian Infant with Pearson Marrow Syndrome. *Iranian Journal of Pediatrics*, *1*, 107–112.

Atale, A., Bonneau-Amati, P., Rötig, A., Fischer, A., Perez-Martin, S., de Lonlay, P., Niaudet, P., De Parscau, L., Mousson, C., Thauvin-Robinet, C., Munnich, A., Huet, F., & Faivre, L. (2009). Tubulopathy and pancytopaenia with normal pancreatic function: A variant of Pearson syndrome. *European Journal of Medical Genetics*, *52*(1), 23–26. https://doi.org/10.1016/j.ejmg.2008.10.003

Ayed, I. Ben, Chamkha, I., Mkaouar-Rebai, E., Kammoun, T., Mezghani, N., Chabchoub, I., Aloulou, H., Hachicha, M., & Fakhfakh, F. (2011). A Tunisian patient with Pearson syndrome harboring the 4.977kb common deletion associated to two novel large-scale mitochondrial deletions. *Biochemical and Biophysical Research Communications*, *411*(2), 381–386. https://doi.org/10.1016/j.bbrc.2011.06.154

Baerlocher, K. E., Feldges, A., Weissert, M., Simonsz, H. J., & Rotig, A. (1992). Mitochondrial DNA Deletion in an 8-year-old Boy with Pearson Syndrome. *J. Lnher. Metab. Dis*, *15*, 327–330.

Baertling, F., Meissner, T., Troeger, A., Pillekamp, F., Mayatepek, E., Laws, H. J., & Distelmaier, F. (2014). Granulocyte colony stimulating factor for treatment of neutropenia- associated infection in Pearson syndrome. In *Klinische Padiatrie* (Vol. 226, Issue 3, pp. 190–191). Georg Thieme Verlag. https://doi.org/10.1055/s-0034-1368760

Belgacem, Z. H., Dubois, S. M., Jacoby, E., Martin, P. L., Parikh, S. B., Fleming, M. D., & Agarwal, S. (2023). Successful cord blood transplantation for del7q myelodysplastic syndrome in Pearson marrow pancreas syndrome. In *American Journal of Hematology* (Vol. 98, Issue 12, pp. E376–E379). John Wiley and Sons Inc. https://doi.org/10.1002/ajh.27107

Bernes SM., Bacino Carlos, Prezant Toni R., Pearson Margaret A., WoodTerry S., Fournier Patricia, & FischeI-Ghodsian Nathan. (1993). Identical mitochondrial DNA deletion in mother with progressive external ophthalmoplegia and son with Pearson marrow-pancreas syndrome. *The Journal of Pediatrics*.

Binder, V., Steenpass, L., Laws, H.-J., Ruebo, J., & Borkhardt, A. (2012). A Novel mtDNA Large-Scale Mutation Clinically Exclusively Presenting With Refractory Anemia: Is There a Chance to Predict Disease Progression? *J Pediatr Hematol Oncol*, *34*, 283–292. http://www.mitomap.org/MITOMAP,

Chen, X. Y., Zhao, S. Y., Wang, Y., Wang, D., Dong, C. H., Yang, Y., Wang, Z. H., & Wu, Y. M. (2016). A novel mitochondrial DNA deletion in a patient with Pearson syndrome and neonatal diabetes mellitus provides insight into disease etiology, severity and progression. *Mitochondrial DNA*, *27*(4), 2492–2495. https://doi.org/10.3109/19401736.2015.1033712

Crippa, B. L., Leon, E., Calhoun, A., Lowichik, A., Pasquali, M., & Longo, N. (2015). Biochemical abnormalities in Pearson syndrome. *American Journal of Medical Genetics, Part A*, *167*(3), 621–628. https://doi.org/10.1002/ajmg.a.36939

Cursiefen C., Kuchle M., Scheurlen W., & Naumann G.O.H. (1998). Bilateral Zonular Cataract Associated With the Mitochondrial Cytopathy of Pearson Syndrome. *American Journal of Ophthalmology*, *125*(2), 260–261.

de Vries, D. D., Buzing, C. J. M., Ruitenbeek, W., van der Wouw, M. P. M. E., Sperl, W., Sengers, R. C. A., Trijbels, J. M. F., & van Oost, B. A. (1992). Myopathology and a mitochondrial DNA deletion in the Pearson marrow and pancreas syndrome. *Neuromuscul Disord*, *2*(3), 185–195. https://doi.org/10.1016/0960-8966(92)90005-q

Falcon, C. P., & Howard, T. H. (2017). An infant with Pearson syndrome: a rare cause of congenital sideroblastic anemia and bone marrow failure. *Blood*, *129*(19), 2710–2710. https://doi.org/10.1182/blood-2017-02-766881

Gibson, K. M., Bennett, M. J., Mize, C. E., Jakobs, C., Rotig, A., Munnich, A., Lichter-Konecki, U., & Trefz, F. K. (1992). 3-Methylglutaconic aciduria associated with Pearson syndrome and respiratory chain defects. *J Pediatr*, *121*(6), 940–942. https://doi.org/10.1016/s0022-3476(05)80348-8

Giirgey, A., zalp, I., Riitig, A., Cow, T., Tekinalp, G., Erdem, G., Akciirenl, Z., Caglar, M., & Bakkaloglu, A. (1996). A case of Pearson syndrome associated with multiple renal cysts. *Pediatr Nephrol*, *10*, 637–638.

Giirgeya, A., Rötigb, A., Gürnrüka, F., Cemeroglua, P., Sanalioglua, F., & Altaya, C. (1992). Case Report Pearson’s Marrow-Pancreas Syndrome in 2 Turkish Children. *Acta Haem Atol* , *87*, 206–209.

Hiebert, R. M., Welliver, R. C., & Yu, Z. (2016). Fusarium osteomyelitis in a patient with pearson syndrome: Case report and review of the literature. *Open Forum Infectious Diseases*, *3*(4). https://doi.org/10.1093/ofid/ofw183

Jacobs, L. J. A. M., Jongbloed, R. J. E., Wijburg, F. A., De Klerk, J. B. C., Geraedts, J. P. M., Nijland, J. G., Scholte, H. R., De Coo, I. F. M., & Smeets, H. J. M. (2004). Pearson syndrome and the role of deletion dimers and duplications in the mtDNA. *J.Inherit.Metab.Dis.*, *27*.

Muraki K, Nishimura  S, Goto Y, Nonaka I, Sakura N, & Ueda K. (1997). The association between haematological manifestation and mtDNA deletions in Pearson syndrome. *J Inherit Metab Dis*, *20*(5), 697–703. https://doi.org/10.1023/a:1005378527077

Kapsa, R., Thompson, G. N., Thorburn, D. R., Dahl, H.-H. M., Makzuki, S., Byline, E., & Bloi¢, R. B. (1994). A Novel mtDNA Deletion in an Infant with Pearson Syndrome. In *J. Inher. Metab. Dis* (Vol. 17).

Kasbekar, S. A., Gonzalez-Martin, J. A., Shafiq, A. E., Chandna, A., & Willoughby, C. E. (2013). Corneal endothelial dysfunction in Pearson syndrome. *Ophthalmic Genetics*, *34*(1–2), 55–57. https://doi.org/10.3109/13816810.2011.610862

Kefala-Agoropoulou, K., Roilides, E., Lazaridou, A., Karatza, E., Farmaki, E., Tsantali, H., Augoustides-Savvopoulou, P., & Tsiouris, J. (2007). Pearson syndrome in an infant heterozygous for C282Y allele of HFE gene. *Hematology*, *12*(6), 549–553. https://doi.org/10.1080/10245330701400900

Khasawneh, R., Alsokhni, H., Alzghoul, B., Momani, A., Abualsheikh, N., Kamal, N., & Qatawneh, M. (2018). A Novel Mitochondrial DNA Deletion in Patient with Pearson Syndrome. *Medical Archives (Sarajevo, Bosnia and Herzegovina)*, *72*(2), 148–150. https://doi.org/10.5455/medarh.2018.72.148-150

kleinle S., Weismann U, Superti-Furga A, Krahenbuhl S., Boltshauser E., Reichen J., & Liechti-Gallati S. (1997). Detection and characterization of mitochondrial DNA rearrangementsin Pearson and Kearns-Sayre syndromes by long PCR. *Hum Genet*, *100*.

Knerr, I., Metzler, M., Niemeyer, C. M., Holter, W., Gerecke, A., Baumann, I., Trollmann, R., & Repp, R. (2003). Hematologic Features and Clinical Course of an Infant With Pearson Syndrome Caused by a Novel Deletion of Mitochondrial DNA. In *J Pediatr Hematol Oncol •* (Vol. 25, Issue 12).

Köklü, S., Alioǧlu, B., Akbal, E., & Koçak, E. (2010). Celiac disease in siblings with pearson syndrome. *American Journal of the Medical Sciences*, *339*(4), 392–394. https://doi.org/10.1097/MAJ.0b013e3181cefba3

Krahenbtihl, S., Kleinle, S., Henz, S., Leibundgut, K., Liechti, S., Zimmermann, A., & Wiesmann, U. (1999). Microvesicular steatosis, hemosiderosis and rapid development of liver cirrhosis in a patient with Pearson’s syndrome. *Journal of Hepatology*, *31*, 550–555.

Krauch, G., Wilichowski, E., Schmidt, K. G., & Mayatepek, E. (2002). Pearson marrow-pancreas syndrome with worsening cardiac function caused by pleiotropic rearrangement of mitochondrial DNA. *American Journal of Medical Genetics*, *110*(1), 57–61. https://doi.org/10.1002/ajmg.10410

Lacbawan, F., Tifft, C. J., Luban, N. L. C., Schmandt, S. M., Guerrera, M., Weinstein, S., Pennybacker, M., & Wong, L.-J. C. (2000). Clinical Heterogeneity in Mitochondrial DNA Deletion Disorders: A Diagnostic Challenge of Pearson Syndrome. In *J. Med. Genet* (Vol. 95).

Lee, H. F., Lee, H. J., Chi, C. S., Tsai, C. R., Chang, T. K., & Wang, C. J. (2007). The neurological evolution of Pearson syndrome: Case report and literature review. *European Journal of Paediatric Neurology*, *11*(4), 208–214. https://doi.org/10.1016/j.ejpn.2006.12.008

Li C. H., Lam C. W., Lee C. W. A., Kwong N. S., & Szeto S. C. (2003). Pearson’s syndrome: a rare cause of non-immune hydrops fetalis. *Chinese Medical Journal*, *116*(13), 1952–1954.

Lichter-Konecki, U., Trefz, F. K., Rotig, A., Munnich, A., Pfeil, A., & Bremer, H. J. (1992). *3-Methylglutaconic aciduria in a patient with Pearson syndrome*.

Liu, R., Mo, G. L., & Song, Y. Z. (2021). Identification of a novel large deletion of the mitochondrial DNA in an infant with Pearson syndrome: a case report. *Translational Pediatrics*, *10*(1), 204–208. https://doi.org/10.21037/tp-20-138

Lohi, O., Kuusela, A. L., & Arola, M. (2005). A novel deletion in a Pearson syndrome infant with hypospadias and cleft lip and palate. *Journal of Inherited Metabolic Disease*, *28*(6), 1165–1166. https://doi.org/10.1007/s10545-005-0075-0

McShane MA, Hammans SR, Sweeney M, Holt IJ, Beattie TJ, & Brett EM. (1991). Pearson syndrome and mitochondrial encephalomyopathy in a patient with a deletion of mtDNA. *Am J Hum Genet*, *48*(1), 39–42.

Maguluri, S., & Recchia, F. M. (2007). PARAFOVEOLAR INTRARETINAL CRYSTALS IN PEARSON SYNDROME. *RETINAL CASES & BRIEF REPORTS*, *1*, 239–240.

Mats, S. J., & Cortez, D. (2020). Pearson marrow-pancreas syndrome with cardiac conduction abnormality necessitating prophylactic pacemaker implantation. *Annals of Noninvasive Electrocardiology : The Official Journal of the International Society for Holter and Noninvasive Electrocardiology, Inc*, *25*(1). https://doi.org/10.1111/ANEC.12681

Momont AC, & Trobe JD. (2009). Transient Corneal Edema and Left Hemisphere Dysfunction in Pearson Syndrome. *J Neuro-Ophthalmol*, *29*(2), 158–159.

Morel, A. S., Joris, N., Meuli, R., Jacquemont, S., Ballhausen, D., Bonafé, L., Fattet, S., & Tolsa, J. F. (2009). Early neurological impairment and severe anemia in a newborn with Pearson syndrome. *European Journal of Pediatrics*, *168*(3), 311–315. https://doi.org/10.1007/s00431-008-0756-4

Morikawa, Y., Matsuura, N., Kakudo, K., Higuchi, R., Koike, M., & Kobayashi, Y. (1993). Virchows Archiv A Pathological Anatomy and Histopathology Pearson’s marrow/pancreas syndrome: a histological and genetic study. In *Virchows Archiv A Pathol Anat* (Vol. 423).

Muraki K., Goto Y, Nishino I, Hayashidani M, Takeuchi S, Horai S, Sakura N, & Ueda K. (1997). Severe lactic acidosis and neonatal death in Pearson syndrome. *J. Inher. Metab. Dis*.

Niaudetl, P., Heidetl, L., Munnichl, A., Schmitzl, J., Bouissou, F., Gubler, M. C., & Rotigl, A. (1994). Pediatric Nephrology Deletion of the mitochondrial DNA in a case of de Toni-Debr-Fanconi syndrome and Pearson syndrome. In *Pediatr Nephrol* (Vol. 8).

Nilay, M., & Phadke, S. R. (2020). Pearson Syndrome: Spontaneously Recovering Anemia and Hypoparathyroidism. *Indian Journal of Pediatrics*, *87*(12), 1070–1072. https://doi.org/10.1007/s12098-020-03333-9

Park, J., Ryu, H., Jang, W., Chae, H., Kim, M., Kim, Y., Kim, J., Lee, J. W., Chung, N. G., Cho, B., & Suh, B. K. (2015). Novel 5.712 kb mitochondrial DNA deletion in a patient with Pearson syndrome: A case report. *Molecular Medicine Reports*, *11*(5), 3741–3745. https://doi.org/10.3892/mmr.2014.3127

Reddy, J., Jose, J., Prakash, A., & Devi, S. (2019). Pearson syndrome: a rare inborn error of metabolism with bone marrow morphology providing a clue to diagnosis. *Sudanese Journal of Paediatrics*, 161–164. https://doi.org/10.24911/sjp.106-1534158413

Ribes, A., Riudor, E., Valcarel, R., Salva, A., Castello, F., Murillo, S., Dominguez, C., & Jakobs, C. (1993). Pearson Syndrome: Altered Tricarboxylic Acid and Urea-Cycle Metabolites, Adrenal Insufficiency and Corneal Opacities. In *J. Inher. Metab. Dis* (Vol. 16).

Rötig, A., Bourgeron, T., Chretien, D., Rustin, P., & Munnich, A. (1995). Spectrum of mitochondrial DNA rearrangements in the Pearson marrow-pancreas syndrome. *Hum Mol Genet*, *4*(8), 1327–1330. https://doi.org/10.1093/hmg/4.8.1327

Santorelli, F. M., Barmada, M. A., Pons, R., Zhang, L. L., & DiMauro, S. (1996). Leigh-type neuropathology in Pearson syndrome associated with impaired ATP production and a novel mtDNA deletion. *Neurology*, *47*(5), 1320–1323. https://doi.org/10.1212/wnl.47.5.1320

Sato, T., Muroya, K., Hanakawa, J., Iwano, R., Asakura, Y., Tanaka, Y., Murayama, K., Ohtake, A., Hasegawa, T., & Adachi, M. (2015). Clinical manifestations and enzymatic activities of mitochondrial respiratory chain complexes in Pearson marrow-pancreas syndrome with 3-methylglutaconic aciduria: a case report and literature review. *European Journal of Pediatrics*, *174*(12), 1593–1602. https://doi.org/10.1007/s00431-015-2576-7

Seneca, S., De Meirleir, L., De Schepper, J., Balduck, N., Jochmans, K., Liebaers, I., & Lissens, W. (1997). Pearson marrow pancreas syndrome: a molecular study and clinical management. *Clin Genet*, *51*(5), 338–342. https://doi.org/10.1111/j.1399-0004.1997.tb02484.x

Shanske, S., Tang, Y., Hirano, M., Nishigaki, Y., Tanji, K., Bonilla, E., Sue, C., Krishna, S., Carlo, J. R., Willner, J., Schon, E. A., & DiMauro, S. (2002). Identical mitochondrial DNA deletion in a woman with ocular myopathy and in her son with pearson syndrome. *Am J Hum Genet*, *71*(3), 679–683. https://doi.org/10.1086/342482

Shoeleh, C., Donato, U. M., Galligan, A., & Vitko, J. (2023). A Case Report on Pearson Syndrome With Emphasis on Genetic Screening in Patients Presenting With Sideroblastic Anemia and Lactic Acidosis. *Cureus*. https://doi.org/10.7759/cureus.33963

Son, J. S., Seo, G. H., Kim, Y. M., Kim, G. H., Jin, H. K., Bae, J. S., Im, H. J., Yoo, H. W., Lee, B. H., & Alqasim, A. M. Z. (2022). Clinical and genetic features of four patients with Pearson syndrome: An observational study. *Medicine (United States)*, *101*(5), 28793. https://doi.org/10.1097/MD.0000000000028793

Stoddard, R. A., McCurnin, D. C., Shultenover, S. J., Usaf, M., Wright, J. E., & deLemos, R. A. (1981). Syndrome of refractory sideroblastic anemia with vacuolization of marrow precursors and exocrine pancreatic dysfunction presenting in the neonate. *Brief Clinical and Laboratory Observations* , *99*(2).

Superti-Furga, A., Schoenle, E., Tuchschmid, P., Caduff, R., Sabato, V., DeMattia, D., Gitzelmann, R., & Steinmann, B. (1993). Pearson bone marrow-pancreas syndrome with insulin-dependent diabetes, progressive renal tubulopathy, organic aciduria and elevated fetal haemoglobin caused by deletion and duplication of mitochondrial DNA. *Eur J Pediatr*, *152*(1), 44–50. https://doi.org/10.1007/bf02072515

Tedjaseputra, A., Radhakrishnan, K., Martin, M., Downie, P., Stergiotis, M., & Bain, B. J. (2023). Pearson syndrome. In *American Journal of Hematology* (Vol. 98, Issue 3, pp. 527–528). John Wiley and Sons Inc. https://doi.org/10.1002/ajh.26794

Tesarova, M., Vondrackova, A., Stufkova, H., Veprekova, L., Stranecky, V., Berankova, K., Hansikova, H., Magner, M., Galoova, N., Honzik, T., Vodickova, E., Stary, J., & Zeman, J. (2019). Sideroblastic anemia associated with multisystem mitochondrial disorders. *Pediatric Blood and Cancer*, *66*(4). https://doi.org/10.1002/pbc.27591

Topaloğlu, R., Lebre, A. S., Demirkaya, E., Kuşkonmaz, B., Coşkun, T., Orhan, D., Gürgey, A., & Gümrük, F. (2008). Two new cases with Pearson syndrome and review of Hacettepe experience. In *The Turkish Journal of Pediatrics* (Vol. 50).

Toth T, Bokay J, Szijnyi L, Nagy B, & Papp Z. (1998). CLINICAL GENEXICS Detection of mtDNA deletion syndrome by two independent from Guthrie card. In *Clin Gmcr* (Vol. 53).

Wild, K. T., Goldstein, A. C., Muraresku, C., & Ganetzky, R. D. (2020). Broadening the phenotypic spectrum of Pearson syndrome: Five new cases and a review of the literature. *American Journal of Medical Genetics, Part A*, *182*(2), 365–373. https://doi.org/10.1002/ajmg.a.61433
